# Effects of UV-C Irradiation and Vacuum Sealing on the Shelf-Life of Beef, Chicken and Salmon Fillets

**DOI:** 10.3390/foods12030606

**Published:** 2023-02-01

**Authors:** Asrar Nabil Damdam, Ashwaq Alzahrani, Lama Salah, Kahled Nabil Salama

**Affiliations:** 1 Sensors Lab, Advanced Membranes and Porous Materials Center, Computer, Electrical and Mathematical Science and Engineering Division, King Abdullah University of Science and Technology (KAUST), Thuwal 23955-6900, Saudi Arabia; 2 Uvera Lab, Research and Development Department, Uvera Inc., Thuwal 23955-6900, Saudi Arabia

**Keywords:** shelf-life, food waste, UV-C irradiation, vacuum packaging, meats preservation, chicken preservation, beef preservation, salmon preservation

## Abstract

One-third of the world’s food supply is lost, with meat being a major contributor to this loss. Globally, around 23% of all meat and 35% of all seafood products are lost or wasted. Meats and seafood products are susceptible to microbial spoilage during processing, storage, and distribution, where microbial contamination causes significant losses throughout the supply chain. This study examined the efficacy of UV-C irradiation and vacuum-sealing in preventing microbiological deterioration in beef, chicken, and salmon fillets. The samples were sterilized using a constant UV-C irradiation dose of 360 J/m^2^ and stored under a reduced pressure of 40 kPa. A microbiological analysis was conducted daily to examine the microbial contamination, which included counting the colonies of *Pseudomonas* spp., aerobic bacteria, lactic acid bacteria (LAB), *Salmonella*, and *Escherichia coli*, as well as monitoring the increase in pH levels. The results demonstrated a statistically significant difference (*p* > 0.05) in the aerobic bacteria counts between the storage conditions and storage days in all samples, which is a primary indicator of microbial spoilage. In contrast, the differences varied in the *Pseudomonas* spp. and LAB counts between the storage conditions and storage days, and there was no significant difference (*p* < 0.05) in the pH levels between the storage conditions. The results indicate that the combination of UV-C irradiation and vacuum sealing effectively inhibits microbial growth and extends the shelf-life of beef, chicken, and salmon fillets by 66.6%.

## 1. Introduction

The growing global population has led to a rise in the generation of food waste. Globally, it is estimated that 1.3 billion tons of food are wasted as a result of post-harvest loss, spoilage, and sensory degradation [1]. Meat and industrial fish waste are significant contributors to food waste. Despite the advancements in refrigeration, chemical preservatives, and the use of modern technologies, it has been estimated that 25% of all food produced worldwide is lost post-harvest or post-slaughter due to microbial spoilage, making this the most prevalent reason for food-quality deterioration and waste [2]. Global meat (including chicken) production was around 340 million tons in 2018; however, it was estimated that 23% of total meat products are wasted throughout the entire supply chain [3,4]. Similarly, seafood (i.e., salmon) production in 2018 was about 178.5 million tons, but up to 35% of total seafood products are lost or wasted [5]. These wastes are often mixed with other waste materials or disposed of directly in the dumpsite. Hence, meat, chicken, and seafood products cause large quantities of waste, which ultimately negatively impacts the environment [1,5].

With consumers’ growing awareness and need for nutritious, fresh, and safe meat products, scientists are working tirelessly to create and discover several creative and progressive food preservation systems for future commercial use [6]. In recent years, a wide variety of innovative thermal and nonthermal meat processing and preservation methods have attracted considerable attention. High-pressure processing (HPP), radiofrequency (RF), pulsed electric field technology (PEF), ozone processing (OP), vacuum packaging (VP), and modified atmosphere packaging (MAP) are among the most common solutions [7,8,9,10,11]. Aside from the conventional preservation techniques, which often include heat or chemical interventions, ultraviolet (UV) irradiation has been emerging for the preservation of perishables, including meat items [12]. Various researchers investigated the effect of different UV-C irradiation doses on the shelf-life of meat and seafood products. For instance, treating chicken breasts using a UV-C dose of 5 KJ/m^2^ resulted in increasing the shelf-life of chicken breasts by up to 6 days [13]. In another study [14] researchers demonstrated that a UV-C dose of 5–15 J/cm^2^ can reduce the *E. coli* in beef by 1.02 log_10_ CFU/mL after only 5 min of exposure, and in chicken and pork by about 1.6 log_10_ CFU/mL after 4 and 10 min of exposure, respectively [14]. Further research successfully extended the shelf-life of fresh skin-on and skinless chicken fillets by 6 and 7 days, respectively, using a UV-C dose of 50 mJ/cm^2^ [15]. Similarly, McLoed et al. [16] examined the impact of a UV-C dose of 3.0 J/cm^2^ on various pathogenic and spoilage bacteria in skinless chicken breasts and found that the microbial loads were generally reduced. However, both studies [15,16] observed a slight burnt odor, which is one of the side effects of using high doses of UV-C irradiation. However, the odor was not observed after cooking the chicken breasts in both cases. Wang et al. [17] studied the impact of a range of UV-C doses (1000–4000 mJ/cm^2^) on chicken breast pathogenic microbes and illustrated that a UV-C dose of 4000 mJ/cm^2^ is effective in reducing the microbial loads of *S. typhimurium*, *E. coli* O157:H7, and *L. monocytogenes* with no sensory quality degradation, such as pH, texture, or color, indicating that high UV-C irradiation doses could be utilized to extend the shelf-life of chicken by a few days [17]. In addition, Lázaro et al. [18] demonstrated that a UV-C dose of 1.95 mW/cm^2^ could substantially reduce the bacterial load of *Salmonella*, total aerobic mesophilic bacteria, and *Enterobacteriaceae,* and enhance the shelf life of chicken breasts by up to 9 days. Another study evaluated the effectiveness of a 0.0075–0.6 J/cm^2^ UV-C irradiation dose in reducing the microbial loads in raw and smoked salmon and found that the shelf-life was increased by up to 7 days for raw salmon and 14 days for smoked salmon [19].

Aside from UV-C irradiation, vacuum packaging is one of the most-utilized non-thermal meats preservation methods. It can extend the shelf life of various meat, poultry, and seafood products by 50–400%, which results in a reduction in losses, enabling the distribution of goods over vast distances, and aiding in the maintenance of product quality [20]. Duran and Kahve [21] found that vacuum-sealing beef that is stored at 4 °C can prevent the growth of mesophilic aerobic bacteria, *Staphylococcus aureus*, and lactic acid bacteria, extending the shelf-life of beef for 7–15 days. Other researchers investigated the effect of using low-density polyethylene vacuum bags on the shelf-life of chicken and reported a shelf-life increase of 15 days [22]. Gertzou et al. [23] noted that the shelf-life of fresh chicken legs can be extended for 10 days if packed in polyamine/polyethylene bags using vacuum sealing. Additionally, the use of vacuum sealing for salmon preservation has shown promising results. For instance, Chan et al. [24] observed that vacuum-sealing using polyethylene terephthalate containers can increase the shelf-life of salmon fillets by up to 18–20 days, which is 1.5 times higher than non-packaged fillets. However, in the case of fresh salmon loins stored at 5 °C, Fidalgo et al. [25] demonstrated that the vacuum-sealing using polyamide-polyethylene bags can keep total aerobic mesophilic count at an acceptable level for at least 15 days. In extended research [26], the same researchers concluded that the lipid and physical profiles of fresh salmon loins remain under acceptable levels for 15 days using polyamide-polyethylene bags. These results demonstrate the positive impact of vacuum-sealing on the shelf-life of salmon. The singular use of UV-C irradiation technology or vacuum-sealing technology has been widely researched and implemented in numerous food industries. However, the effect of combining both preservation technologies on the shelf-life of meat products has not been investigated to date. Therefore, this research examines the efficacy of combining UV-C irradiation with vacuum sealing in increasing the shelf-life of meats, as compared to the usage of UV-C irradiation or vacuum sealing alone. Beef, chicken, and salmon fillets were selected for this study due to their high market demand, economic value, short shelf-life, and carbon footprint. The consumption of meats has increased by 42.7% globally in the last 20 years, reaching 328.4 million metric tonnes in 2021 [27]. The livestock industry is a major contributor to climate change, accounting for 12–18% of all GHG emissions [28]. However, it was estimated that 23% of meat is wasted throughout the entire supply chain [3]. Therefore, it is important to explore solutions that can reduce the wastage of meat products throughout the supply chain to limit the overall environmental impact of its production and consumption.

## 2. Materials and Methods

### 2.1. Sample Preparation

Fresh and pre-packed beef, chicken, and salmon were purchased from a local supermarket (Thuwal, Saudi Arabia). The cuts of meat samples were CAB boneless rib beef, boneless chicken breast, and salmon lion. The weights of the cuts ranged from 120 to 150 g for the beef, 120 to 150 g for the chicken, and 550 to 520 g for the salmon. The samples were transferred to the laboratory within 15 min of purchase and kept in thermal bags during transport.

### 2.2. Experimental Setups

A sterile knife was used to cut the meats into 2 × 2 cm cubes immediately upon purchase. The shelf-life experiment consisted of four beef samples, each with a weight of 150 g, four salmon samples, each with a weight of 130 g, and four chicken samples, each with a weight of 120 g. All the samples were stored in 1.4 L Eastman Tritan PCTG TX1001 plastic containers at a humidity level of 60% and a temperature of 4 °C. To study the effectiveness of the combination of UV-C irradiation and vacuum-sealing on the shelf-life of various types of meat, each of the four samples from the three meat categories was stored in a distinct storage environment, as follows:

(1) A sterilized and anaerobic environment created using UV-C irradiation and vacuum-sealing (UV-C and vacuum);

(2) A sterilized aerobic environment created using UV-C-irradiation (UV-C);

(3) An anaerobic environment created by vacuum-sealing (vacuum);

(4) A normal aerobic and unsterilized environment (control).

**Figure 1:**
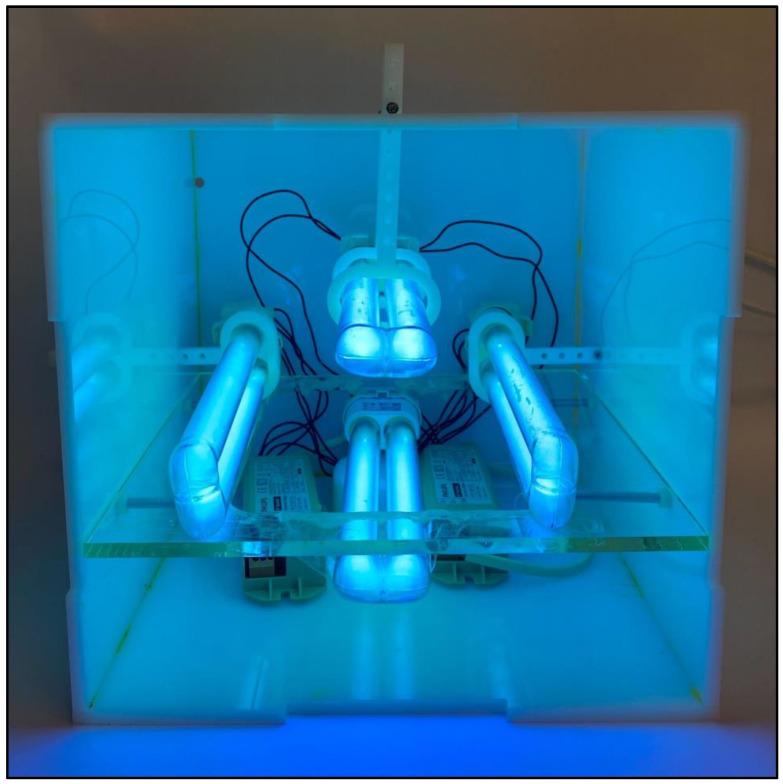
The UV-C irradiation setup.

To validate the results of the microbiological quantification and ensure the accuracy of the shelf-life estimation, two shelf-life experimental runs were conducted for each meat type and storage condition, rustling in a total of 24 samples. Each was stored in a separate container. The samples in both runs contained the same meat cuts, weights, date of purchase, labeled expiry date, and preparation methods. For the experimental setups, four UV-C lamps (2G11, 253.7 nm, 18 W, Philips, Shanghai, China) were mounted on movable racks to expose the meat samples in the containers to light from the left, right, bottom, and top sides, as shown in Figure 1. The average distance between the samples and each light bulb was 2.45 cm and the exposure period was 30 s. A constant UV-C irradiation dose of 360 J/m^2^ was used in this study, and the UV-C light intensity was measured using an ultraviolet meter (Zenith, Atlantic Ultraviolet Corporation, New York, NY, USA). After the UV-C light exposure, the storage containers were vacuum-sealed using an a12-volt oxygen vacuum pump (JP1) until the pressure was reduced to 40 kPa. The oxygen levels in the vacuumed containers ranged between 1.7% and 1.8%, while they ranged between 20.7% and 21% in the containers that were not vacuumed, according to the AR8100 oxygen sensor.

### 2.3. Shelf Life and Quality Examination

#### 2.3.1. Microbial Analysis

ISO standards were followed to count the populations of *Pseudomonas* spp. (ISO 13720:2010), lactic acid bacteria (ISO 13721:1995), and aerobic mesophilic bacteria (ISO 4833-2:2013) in all samples by applying Pseudomonas CFC Agar Base (Oxoid, Thermo Scientific, Basingstoke, UK), along with SR0103E Pseudomonas C-F-C supplement and glycerol, De Man, Rogosa Sharpe agar (MRS, Oxoid, Thermo Scientific, Basingstoke, UK) and Plate Count Agar (PCA, Oxoid, Thermo Scientific, Basingstoke, UK), respectively. In addition, *Salmonella* spp. was only detected (ISO 6579:2002) in the beef and chicken samples, while *Escherichia coli* was only detected (ISO 7251:2005) in the salmon samples. Rappaport Vassiliadis soya peptone broth (RVS, Condalab, Madrid, Spain), Muller–Kauffmann Tetrathionate Novobiocin Broth (MKTTn, Condalab, Madrid, Spain) and Xylose Lysine Deoxycholate (XLD, Condalab, Madrid, Spain) were used to detect *Salmonella* spp., whereas Lauryl Sulfate Tryptose Broth (LST, Oxoid, Thermo Scientific, Basingstoke, UK) and *E. coli* Broth (EC, Oxoid, Thermo Scientific, Basingstoke, UK) were used to detect *E. coli* using the most probable number (MPN) method. The workspace and equipment were sterilized using 70% alcohol. After that, for the serial dilutions, 3 g of each sample were put in stomacher bags and 27 mL of sterile physiological solution was added and homogenized for 2 min in the stomacher to create the first suspension (10^−1^ dilution). A sterile pipette was used to add 1 mL from the first dilution to 9 mL of the physiological solution for the second dilution (10^−2^). More dilutions were created, following the same procedures. Then, 100 µL of each dilution was spread-plated into CFC to count the population of pseudomonas sp., while pour-plating 1 mL from every dilution into PCA and MRS to count the populations of aerobic mesophilic bacteria and LAB. The Petri-dishes of PCA and MRS were incubated at 30 °C for 72 h while CFC Petri-dishes were incubated for 48 h at 25 °C. The *Salmonella* sp. was detected by incubating the first dilution for a period not exceeding 20 h, enriching 0.1 mL into RVS broth, followed by a 24-h incubation at 41.5 °C. In addition, 1 mL was enriched into MKTTn broth and incubated for 24 h at 37 °C. After that, 10 µL of each broth was streaked on XLD Petri dishes and then incubated for 24 h at 37 °C. To count the population of *E. coli*, 3 tubes containing Durham tube and LST broth were used for each of the 10^−1^ 10^−2^, and 10^−3^ dilutions. A total of 1 mL was taken from the dilutions into the RVS tube and incubated for 24 h at 37 °C. Next, 10 µL was taken from each RVS tube that showed positive results based on the appearance of growth and gas production in Durham tubes and placed in EC broth, and then incubated for 24 h at 45.5 °C. Microbial analyses were conducted daily throughout the shelf-life of each sample to examine the impact of each storage condition on the growth rate, which affects the sensory properties of the samples. A colony-forming units per gram (CFU/g) was used to analyze the results.

#### 2.3.2. pH measurement

Thermo Scientific Orion 5 Star pH meter was used to monitor the pH levels. Following the ISO 2917:1999 guidelines, 5 g of each sample were taken, and then the pH was measured directly from the sample at 20 ± 2 °C.

#### 2.3.3. Statistical Analysis

A one-way analysis of variance (ANOVA) was performed to determine the statistical significance (*p* < 0.05) of the differences between storage conditions using SPSS Statistics software (SPSS Inc, Chicago, IL, USA). Pairwise comparison Tukey HSD test was used to identify the significant differences (*p* < 0.05) in the storage conditions and microbial counts.

## 3. Results

### 3.1. Microbial Analysis

A microbiological analysis is essential to determine the efficacy of each storage condition in reducing the total viable counts of microbes that contribute to the sensory degeneration and spoiling of meats. The variations in the total viable counts of *Pseudomonas* spp., LAB, and aerobic bacteria in beef, chicken, and salmon samples under various storage conditions are shown in Table 1, Table 2 and Table 3, respectively.

#### 3.1.1. Beef

The results of the one-way ANOVA analysis and Tukey HSD test of the microbial counts of *Pseudomonas* spp., LAB, and aerobic mesophilic bacteria for the beef are shown in Table 1. The *Pseudomonas* spp. results showed a significant difference (*p* < 0.05) in their growth rate and the number of colonies in the UV-C and Vacuum storage condition in comparison with the other storage conditions, where a lower growth rate and a smaller number of colonies was achieved in the UV-C and Vacuum storage condition. On the contrary, the LAB results indicated no difference (*p* > 0.05) in growth rate under the four storage conditions on days 1, 2, 3, and 5. On day 4, the LAB stored under UV-C and vacuum conditions had a different superscript letter, indicating that the count was lowest on that day compared to the other storage conditions. As for the aerobic bacteria, Table 1 shows that the superscript letters of the vacuum storage, UV-C storage, and UV-C and Vacuum storage were the same from the first to the fourth day, but different from the control storage. The UV-C and Vacuum treatment had a lower average number of aerobic bacteria than the other treatments. *Salmonella* spp. was detected daily; however, no colonies were found in any beef sample throughout the experimental period. Although all treatments other than the control were more efficient in limiting the growth of aerobic bacteria, the results of the Tukey HSD test suggest that the UV-C and Vacuum storage condition is the most effective when compared to other storage conditions.

#### 3.1.2. Chicken

Table 2 demonstrates the results of the ANOVA analysis and Tukey HSD test of the chicken samples. As shown in Table 2, the superscript letters and numbers are distinct for each storage condition and storage day. In the test results of *Pseudomonas* spp., there were lower average values in the vacuum storage and the UV-C and Vacuum storage, which indicate that the two treatments were more effective than the other treatments in inhibiting the growth of *Pseudomonas* spp. In contrast, on the second, third, and fifth days, distinct superscript letters were observed in the vacuum alone, the UV-C and Vacuum, and the control, showing that these two treatments were still more successful than the control. As for the LAB, the UV-C and Vacuum treatment was still the most effective method for limiting the LAB growth as it shared the same superscript letter with the other storage conditions until the fourth day. Regarding the aerobic bacteria, the Tukey HSD test revealed that the vacuum alone, UV-C only, and UV-C and Vacuum treatments shared the same superscript letter from the first to the fifth day, which indicates that these three storage conditions were equally efficient at inhibiting the growth of aerobic bacteria in comparison to the control condition. Nonetheless, it can be observed that there is a change in the superscript number between the three treatments, which indicates a higher growth rate in the UV-C storage and the vacuum storage in comparison with the UV-C and Vacuum storage condition. *Salmonella* spp. colonies were detected on day 3 in the first run of the vacuum sample and in both of the control samples; hence, the vacuum sample in the first run was considered expired. It is worth mentioning that no *Salmonella* colonies were detected in any of the UV-C-irradiated samples throughout the entire experimental period. Based on these findings, the UV-C and Vacuum storage condition remains the most efficient for inhibiting the growth of all the bacteria throughout the experimental period.

#### 3.1.3. Salmon

The influence of the four storage conditions on the microbial growth of the salmon samples was investigated, and the results are shown in Table 3. The analysis revealed that the vacuum storage is more efficient than the UV-C storage in inhibiting the growth of *Pseudomonas* spp, whereas the UV-C and Vacuum storage had the lowest average count of *Pseudomonas* spp. from the first to the fifth day, indicating that this was the most effective when compared to other treatments. For LAB bacteria, all storage conditions had the same superscript letter (a) and number (1), which indicates no significant difference (*p* > 0.05) and reveals that all the storage conditions had the same effect on the growth of LAB throughout the experimental period. Meanwhile, the aerobic bacteria exhibited a comparable effect to the *Pseudomonas* spp., with the UV-C and Vacuum treatment never having shared the same superscript letter as the control since the first day. As a result, the combination of UV-C and Vacuum has a considerable influence on the suppression of *Pseudomonas* spp. and aerobic bacteria growth in salmon samples. As for *Escherichia coli*, none of the salmon samples tested positive throughout the experimental periods under all storage conditions.

### 3.2. pH Analysis

The ANOVA and Tukey HSD analyses for the beef, chicken, and salmon samples are illustrated in Table 4. As shown in the beef section, the results reveal that all types of treatments have the same superscript letter, and all treatment days have the same superscript number, indicating no significant difference (*p* > 0.05) in the mean pH of beef across the storage conditions and storage days. As a result, it can be stated that UV-C irradiation and vacuum sealing have no influence on the pH of beef. However, distinct findings were obtained in the Tukey HSD test results on chicken, where multiple different superscripts were detected for both treatment types and treatment days. The chicken results show that, on the first day, all treatments had the same superscript letter which indicates that there was no difference (*p* > 0.05) in the average pH of chicken between the storage conditions on the first day of treatment. On the second day, it was found that the chicken samples stored under UV-C and Vacuum conditions had different superscript letters than the vacuum condition, indicating a significant difference (*p* < 0.05) in their average pH levels. However, the average pH of chicken treated with UV-C irradiation and vacuum-sealing was lower than the pH of samples stored under vacuum conditions, indicating that the use of UV-C irradiation simultaneously with vacuum-sealing was more effective on the second day than just using vacuum-sealing. A comparison of the results on the third, fourth and fifth days showed similar results to the first day, where there was no difference in superscript letters between treatments, which indicates that there was no difference in the average pH in the chicken samples between treatments after the second day. The pairwise comparison results also showed that, in the control and vacuum-sealing-only treatments, there was no difference in the superscript number, which indicates that, during the 5 days of storage, there was no significant change in the pH of chickens in the two treatments. However, the UV-C and UV-C and Vacuum conditions showed a significant change in the pH of the chicken samples after the third day, as indicated by the different superscript numbers. As for the salmon samples, the Tukey HSD analysis indicates that there was no difference in the superscript letters between the samples stored under control, vacuum, UV-C, and UV-C and Vacuum conditions on days 1, 2, 3, 4, and 5. In contrast, only the vacuum sealing alone and UV-C and Vacuum treatments exhibited variations in the superscript number, while the control and UV-C-irradiation-only treatments exhibited no alterations in the superscript number. Hence, it can be concluded that neither the control nor UV-C irradiation alone significantly altered the pH of salmon.

## 4. Discussion

In this research, the expiration of the meat samples was determined following the microbiological criteria of foodstuff of the GCC Standardization Organization (GSO 1016:2015). Based on the GSO criteria, the growth of aerobic bacteria is the key indicator of the expiration of meats, where a sample is deemed spoiled when the population count of aerobic bacteria reaches the threshold of 10^6^ CFU/g in row beef and fish and 5 × 10^6^ CFU/g in chicken. Another indicator of the expiration of chicken and beef is the presence of *Salmonella*, where the detection of a single *Salmonella* colony is enough to reject the sample. For salmon, the appearance of *E. coli* is another expiration indicator. However, a salmon sample is deemed spoiled if the growth of aerobic bacteria reaches the threshold of 10^6^ CFU/g, even in the absence of *E. coli*.

**Figure 2:**
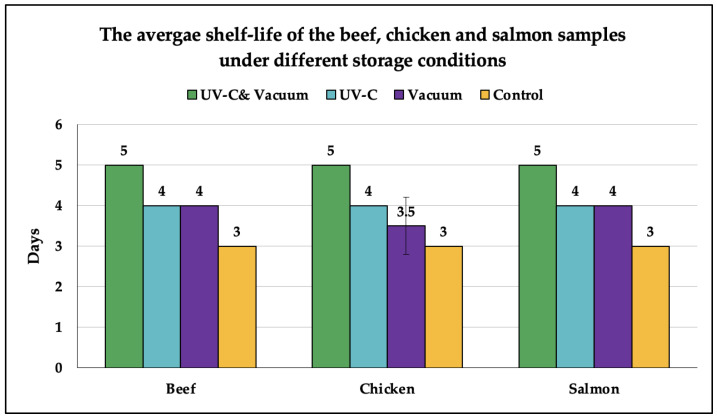
The shelf-life of the beef, chicken, and salmon fillet samples stored under different conditions (days). The standard deviations of the two shelf-life measurement runs were calculated and used to derive error bars.

Figure 2 shows the average shelf-life values that were achieved for the beef, chicken, and salmon fillets under different storage conditions. The results were mainly determined based on the mean values of the aerobic bacteria population counts that were illustrated in Table 1, Table 2 and Table 3, where the beef, chicken, and salmon samples were considered spoiled once they reached the expiration threshold of 10^6^ CFU/g. Nevertheless, based on the Tukey HSD test results, there was a significant difference (*p* < 0.05) between the numerical superscripts on the expiration day and the day just before spoilage for all the samples, which indicates a rapid increase in the microbial growth rate; hence, the occurrence of microbial spoilage. It is worth mentioning that the indicated expiration dates of the control samples matched the dates printed on the packaging of each meat product, validating the precision of the microbiological examination and the results.

Nonetheless, as shown in Figure 2, the standard error of the average shelf life is zero for all samples, except for the chicken under vacuum storage conditions. This was due to the expiration of the chicken sample in the first experimental run, on day 3, as a result of the detection of *Salmonella*. Although the aerobic bacteria population in both chicken runs reached the expiration threshold on day 4, and the Tukey HSD test indicates the spoilage of both samples on day 4, the sample in run 1 was deemed spoiled on day 3 due to the presence of *Salmonella*. Aside from that, *Salmonella* was detected on day 3 in all the control samples. In contrast, no *Salmonella* colonies were detected in any of the UV-C-irradiated samples throughout the entire experimental period, which indicates the sufficiency of the 360 J/m^2^ UV-C irradiation dose for microbial deactivation.

Unlike aerobic bacteria and *Salmonella*, *Pseudomonas* spp. does not pose a significant concern to public health and is mostly known as a spoilage germ rather than a pathogen; therefore, there is no specific safety threshold [29]. However, numerous studies have demonstrated a direct connection between the population of *Pseudomonas* and deteriorations in the sensory qualities of various chilled meat products, which occur when the *Pseudomonas* spp. count ranges between 10^7^ and 10^8^ CFU/g [30,31]. To preserve acceptable sensory characteristics in chilled meats, it is advised to keep the *Pseudomonas* spp. count below the threshold of 10^3^ CFU/g [30]. However, the LAB population of 10^7^ CFU/g is commonly linked with the development of an off-odor and sour flavor in chilled meats [32,33]. However, because LAB are moderately proteolytic, they do not create significant quantities of amines or sulfides, making the unpleasantness of LAB-caused meat quality degradation less of a concern [34]. It is worth mentioning that no changes were observed in the sensory qualities of the samples on their expiration dates in this study. This can be also seen in the *Pseudomonas* spp. and LAB results, where their population counts range between 10^2^ and 10^4^ CFU/g at the end of the shelf-life of all samples, which is significantly below the microbial population counts linked to sensory deterioration in the previously mentioned studies.

The pH measurements were carried out to investigate the effect of each storage condition on meat quality, which is directly linked with microbial growth and shelf-life. Naturally, the acidic environment of the low pH in meat products inhibits microbial growth and lengthens their shelf-life. On the other hand, a higher level of pH in meats would promote the growth of microorganisms, which will shorten the storage life [35]. As illustrated in Table 1, Table 2 and Table 3, the pH levels in the beef, chicken, and salmon samples generally increased, but the rates of increase varied depending on the storage conditions. The pH increase rates could be associated with the microbial growth demonstrated in Table 1, Table 2 and Table 3, which shows the effect of anaerobic storage conditions on slowing down the pH increase rate. Notably, the UV-C and vacuum samples showed the slowest pH growth rate, which confirms the notion that combining UV-C irradiation with vacuum sealing creates an environment that is unfavorable for the growth of bacteria and extends the shelf life of meats.

The shelf-life results in this study are lower than those in other studies that investigated the effect of UV-C irradiation or vacuum packaging on the shelf-life of various meat products, for several different reasons. For instance, another study achieved a shelf-life of 7 days for the skinless chicken breast as a result of UV-C irradiation. However, a higher UV-C irradiation dose range of 50–300 mJ/cm^2^ was used in that research, which caused the samples to develop a burning odor as a side effect [15]. In addition, the vacuum-sealing technique and the type of packaging film play a significant role in determining the shelf life of beef products. Other researchers investigated the effect of low-density polyethylene bags [22], polyamine/polyethylene bags [23], and polyethylene terephthalate containers [24], and achieved a longer shelf-life for different meat products. The microbial spoilage criteria, storage temperature, and pressure also vary among studies, which directly influences the shelf-life results reported by different researchers. Since our objective is to find new and more effective techniques to extend the short shelf-life of perishables, our future research may extend to studying the effects of different packaging materials, storage pressures, and UV-C-irradiation doses on the shelf-life of meat products.

## 5. Conclusions

The efficiency that combining UV-C irradiation and vacuum-sealing has in increasing the shelf-life of beef, chicken, and salmon fillets stored at 4 °C was evaluated and compared with the effect of normal storage, the sole use of UV-C irradiation, and the sole use of vacuum-sealing on the shelf-life of meats. The population count of *Pseudomonas* spp., lactic acid bacteria, and aerobic bacteria, as well as pH analyses, were used to determine the samples’ quality and shelf-life. The anaerobic and sterilized storage environment that was created using UV-C irradiation and vacuum-sealing enhanced the average shelf-life of all the meat categories by 66%. The findings suggest that this storage condition is more efficient for meat preservation than using UV-C irradiation or vacuum-sealing alone, and could greatly minimize the food loss and waste caused by microbial spoilage.

## Figures and Tables

**Table 1:** The results of the one-way ANOVA analysis and Tukey HSD results of the mean microbial counts (CFU/g) of the beef samples.

Bacteria	Day	Control	Vacuum	UV-C	UV-C and Vacuum
*Pseudomonas* spp.	Day 1	(42.40 ± 2.68 ^b1^) × 10^3^	(28.95 ± 6.01 ^a,b1^) × 10^3^	(36.25 ± 5.30 ^b1^) × 10^3^	(18.30 ± 1.41 ^a1^) ×10^3^
	Day 2	(44.55 ± 3.18 ^b1^) × 10^3^	(31.25 ± 5.58 ^a,b1^) × 10^3^	(38.15 ± 6.43 ^a,b1^) × 10^3^	(21.85 ± 3.60 ^a1,2^) × 10^3^
	Day 3	(46.95 ± 3.18 ^b1^) × 10^3^	(35.65 ± 5.86 ^a,b1^) × 10^3^	(42.35 ± 5.44 ^a,b1^) × 10^3^	(26.90 ± 2.96 ^a1,2,3^) × 10^3^
	Day 4	(49.20 ± 2.68 ^b1^) × 10^3^	(39.10 ± 7.21 ^a,b1^) × 10^3^	(44.60 ± 4.94 ^a,b1^) × 10^3^	(29.95 ± 1.06 ^a2,3^) × 10^3^
	Day 5	(16.27 ± 16.01 ^a1^) × 10^4^	(42.85 ± 6.43 ^a1^) × 10^3^	(47.20 ± 4.52 ^a1^) × 10^3^	(33.30 ± 1.41 ^a3^) × 10^3^
LAB	Day 1	(46.10 ± 5.93 ^a1^) × 10^3^	(37.70 ± 3.81 ^a1^) × 10^3^	(40.35 ± 2.33 ^a1^) × 10^3^	(34.85 ± 1.34 ^a1^) × 10^3^
	Day 2	(52.15 ± 7.56 ^a1^) × 10^3^	(40.95 ± 2.61 ^a1^) × 10^3^	(44.30 ± 2.96 ^a1^) × 10^3^	(38.65 ± 2.19 ^a1^) × 10^3^
	Day 3	(17.62 ± 17.64 ^a1^) × 10^4^	(47.40 ± 2.40 ^a1^) × 10^3^	(50.45 ± 2.19 ^a1^) × 10^3^	(45.40 ± 2.68 ^a1^) × 10^3^
	Day 4	(31.70 ± 3.81 ^b1^) × 10^4^	(24.35 ± 4.45 ^b2^) × 10^4^	(30.40 ± 1.41 ^b2^) × 10^4^	(50.80 ± 2.96 ^a1^) × 10^3^
	Day 5	(36.00 ± 3.25 ^a1^) × 10^4^	(27.90 ± 3.53 ^a2^) × 10^4^	(33.90 ± 1.83 ^a2^) × 10^4^	(32.75 ± 6.15 ^a2^) × 10^4^
Aerobic bacteria	Day 1	(35.75 ± 4.31 ^b1^) × 10^4^	(33.90 ± 5.37 ^a1^) × 10^3^	(32.70 ± 4.10 ^a1^) × 10^3^	(13.25 ± 4.45 ^a1^) × 10^3^
	Day 2	(36.35 ± 2.75 ^b1^) × 10^5^	(33.90 ± 2.82 ^a1^) × 10^4^	(32.30 ± 1.27 ^a1^) × 10^4^	(34.60 ± 3.39 ^a1^) × 10^3^
	Day 3	(34.65 ± 2.05 ^b1^) × 10^6^	(33.65 ± 0.63 ^a1^) × 10^5^	(32.30 ± 4.10 ^a1^) × 10^5^	(34.30 ± 2.12 ^a1^) × 10^4^
	Day 4	(34.80 ± 3.39 ^c2^) × 10^7^	(33.10 ± 2.40 ^b2^) × 10^6^	(32.45 ± 1.34 ^b2^) × 10^6^	(32.55 ± 2.05 ^a2^) × 10^5^
	Day 5	(35.40 ± 0.42 ^c3^) × 10^8^	(33.90 ± 1.97 ^b3^) × 10^7^	(33.35 ± 1.06 ^b3^) × 10^7^	(34.55 ± 0.91 ^a3^) × 10^6^

Each value is the mean of two replicates per treatment day, and type of treatment ± standard deviation (SD). For every type of microbe, different superscript letters in the same row indicate significant differences (*p* < 0.05) between storage conditions for the same storage day, and different superscript numbers in the same column indicate a significant difference (*p* < 0.05) between storage day for the same storage condition.

**Table 2:** The results of the one-way ANOVA analysis and Tukey HSD results, with the mean microbial counts (CFU/g) of the chicken samples.

Bacteria	Day	Control	Vacuum	UV-C	UV-C and Vacuum
*Pseudomonas* spp.	Day 1	(43.30 ± 1.27 ^c1^) × 10^3^	(33.20 ± 1.27 ^a,b1^) ×10^3^	(37.10 ± 1.83 ^b,c1^) × 10^3^	(27.05 ± 3.18 ^a1^) × 10^3^
	Day 2	(46.20 ± 0.98 ^b1^) × 10^3^	(36.20 ± 0.98 ^a1,2^) × 10^3^	(39.70 ± 1.83 ^a,b1,2^) × 10^3^	(30.50 ± 3.95 ^a1^) × 10^3^
	Day 3	(48.85 ± 1.20 ^b1^) × 10^3^	(38.60 ± 1.69 ^a1,2^) × 10^3^	(41.65 ± 2.05 ^a,b1,2,3^) × 10^3^	(32.25 ± 3.74 ^a1^) × 10^3^
	Day 4	(50.75 ± 0. 91 ^b1^) × 10^3^	(41.05 ± 3.32 ^a2^) × 10^3^	(44.05 ± 1.48 ^a2,3^) × 10^3^	(34.95 ± 3.88 ^a1^) × 10^3^
	Day 5	(28.90 ± 0.56 ^b2^) × 10^4^	(42.20 ± 1.27 ^a2^) ×1 0^3^	(47.25 ± 0.91 ^a,b3^) × 10^3^	(37.30 ± 3.81 ^a1^) × 10^3^
LAB	Day 1	(38.50 ± 2.40 ^b1^) × 10^3^	(33.90 ± 1.55 ^a,b1^) × 10^3^	(35.70 ± 0.42 ^a,b1^) × 10^3^	(31.85 ± 1.48 ^a1^) × 10^3^
	Day 2	(44.80 ± 1.13 ^b1^) × 10^3^	(38.05 ± 1.06 ^a1,2^) × 10^3^	(40.10 ± 1.13 ^a,b1^) × 10^3^	(37.30 ± 2.68 ^a1,2^) × 10^3^
	Day 3	(49.00 ± 0.00 ^b1^) × 10^3^	(42.20 ± 1.55 ^a2,3^) × 10^3^	(45.20 ± 1.41 ^a,b1^) × 10^3^	(40.80 ± 2.26 ^a2,3^) × 10^3^
	Day 4	(17.15 ± 17.03 ^a1^) × 10^4^	(44.65 ± 1.76 ^a3^) × 10^3^	(27.11 ± 31.52 ^a1^) × 10^3^	(45.65 ± 2.33 ^a3,4^) × 10^3^
	Day 5	(30.35 ± 4.45 ^b1^) × 10^4^	(47.05 ± 1.90 ^a3^) × 10^3^	(50.90 ±0.42 ^a1^) × 10^3^	(50.10 ± 0.70 ^a4^) × 10^3^
Aerobic bacteria	Day 1	(35.65 ± 4.87 ^b1^) × 10^4^	(35.30 ± 5.93 ^a1^) × 10^3^	(32.95 ± 4.59 ^a1^) × 10^3^	(14.80 ± 1.97 ^a1^) × 10^3^
	Day 2	(36.05 ± 3.04 ^b1^) × 10^5^	(35.75 ± 4.17 ^a1^) × 10^4^	(34.25 ± 1.48 ^a1^) × 10^4^	(34.50 ± 4.94 ^a1^) × 10^3^
	Day 3	(37.75 ± 5.16 ^c2^) × 10^6^	(35.15 ± 4.03 ^b2^) × 10^5^	(34.35 ± 4.59 ^a1^) × 10^5^	(35.60 ± 5.37 ^a1^) × 10^4^
	Day 4	(35.85 ± 3.46 ^c2^) × 10^7^	(35.85 ± 3.88 ^b2^) × 10^6^	(34.45 ± 3.74 ^b2^) × 10^6^	(35.50 ± 1.27 ^a1^) × 10^5^
	Day 5	(36.90 ± 2.54 ^b3^) × 10^8^	(34.65 ± 4.17 ^a3^) × 10^7^	(33.45 ± 4.31 ^a3^) × 10^7^	(35.85 ± 3.46 ^a2^) × 10^6^

Each value is the mean of two replicates per treatment day and type of treatment ± standard deviation (SD). For every type of microbe, different superscript letters in the same row indicate significant differences (*p* < 0.05) between storage conditions for the same storage day, and different superscript numbers in the same column indicate a significant difference (*p* < 0.05) between storage day for the same storage condition.

**Table 3:** The results of the one-way ANOVA analysis and Tukey HSD results, with the mean microbial counts (CFU/g) of the salmon samples.

Bacteria	Day	Control	Vacuum	UV-C	UV-C and Vacuum
*Pseudomonas* spp.	Day 1	(21.30 ± 3.39 ^b1^) × 10^3^	(69.50 ± 30.40 ^a,b1^) × 10^2^	(92.50 ± 54.44 ^a,b1^) × 10^2^	(31.50 ± 0.70 ^a1^) × 10^2^
	Day 2	(23.35 ± 3.46 ^c1^) × 10^3^	(81.00 ± 33.94 ^a,b1^) × 10^2^	(14.90 ± 0.42 ^b,c1,2^) × 10^3^	(46.00 ± 2.82 ^a1^) × 10^2^
	Day 3	(25.650 ± 4.03 ^c1^) × 10^3^	(12.050 ± 2.19 ^a,b1^) × 10^3^	(17.600 ± 1.27 ^b,c1,2^) × 10^3^	(56.00 ± 2.82 ^a1^) × 10^2^
	Day 4	(29.35 ± 4.17 ^b1^) × 10^3^	(13.95 ± 2.33 ^a1^) × 10^3^	(19.70 ± 0.84 ^a,b2^) × 10^3^	(88.00 ± 35.35 ^a1,2^) × 10^2^
	Day 5	(31.50 ± 3.81 ^b1^) × 10^3^	(17.05 ± 3.32 ^a1^) × 10^3^	(22.95 ± 0.77 ^a,b2^) × 10^3^	(12.60 ± 0.42 ^a2^) × 10^3^
LAB	Day 1	(31.90 ± 20.50 ^a1^) × 10^2^	(18.40 ± 9.75 ^a1^) × 10^2^	(28.50 ± 22.20 ^a1^) × 10^2^	(15.15 ± 8.83 ^a1^) × 10^2^
	Day 2	(34.70 ± 20.50 ^a1^) × 10^2^	(20.80 ± 10.46 ^a1^) × 10^2^	(30.95 ± 22.55 ^a1^) × 10^2^	(16.65 ± 8.55 ^a1^) × 10^2^
	Day 3	(39.60 ± 20.50 ^a1^) × 10^2^	(25.15 ± 11.38 ^a1^) × 10^2^	(35.05 ± 21.99 ^a1^) × 10^2^	(19.55 ± 10.39 ^a1^) × 10^2^
	Day 4	(15.55 ± 18.02 ^a1^) × 10^3^	(26.85 ± 10.81 ^a1^) × 10^2^	(36.90 ± 21.77 ^a1^) × 10^2^	(22.55 ± 11.66 ^a1^) × 10^2^
	Day 5	(16.78 ± 19.40 ^a1^) × 10^3^	(29.75 ± 10.81 ^a1^) × 10^2^	(26.35 ± 1.48 ^a1^) × 10^2^	(25.45 ± 10.67 ^a1^) × 10^2^
Aerobic bacteria	Day 1	(45.40 ± 3.39 ^b1^) × 10^4^	(37.75 ± 3.88 ^a1^) × 10^3^	(41.75 ± 4.31 ^a1^) × 10^3^	(25.80 ± 9.61 ^a1^) × 10^3^
	Day 2	(48.75 ± 2.33 ^b1^) × 10^5^	(40.70 ± 4.66 ^a1^) × 10^4^	(45.05 ± 2.61 ^a1^) × 10^4^	(43.20 ± 8.34 ^a1^) × 10^3^
	Day 3	(50.55 ± 1.90 ^c1^) × 10^6^	(42.00 ± 1.83 ^a, b1^) × 10^5^	(46.55 ± 3.46 ^b1^) × 10^5^	(31.55 ± 1.06 ^a1^) × 10^4^
	Day 4	(48.30 ± 2.68 ^c2^) × 10^7^	(42.35 ± 0.35 ^b2^) × 10^6^	(47.35 ± 1.76 ^b2^) × 10^6^	(33.65 ± 0.49 ^a2^) × 10^5^
	Day 5	(49.45 ± 2.61 ^b3^) × 10^8^	(42.25 ± 1.20 ^a3^) × 10^7^	(46.15 ± 0.63 ^a3^) × 10^7^	(38.25 ± 2.19 ^a3^) × 10^6^

Each value is the mean of two replicates per treatment day and type of treatment ± standard deviation (SD). For every type of microbe, different superscript letters in the same row indicate significant differences (*p* < 0.05) between storage conditions for the same storage day, and different superscript numbers in the same column indicate a significant difference (*p* < 0.05) between storage day for the same storage condition.

**Table 4:** The one-way ANOVA analysis and Tukey HSD result, with the mean pH values of the beef, chicken, and salmon samples.

Meat Type	Day	Control	Vacuum	UV-C	UV-C andd Vacuum
Beef	Day 1	4.91 ± 0.64 ^a1^	5.41 ± 0.01 ^a1^	5.39 ± 0.02 ^a1^	5.39 ± 0.05 ^a1^
	Day 2	5.45 ± 0.1 ^a1^	5.44 ± 0.01 ^a1^	5.4 ± 0.02 ^a1^	5.41 ± 0.04 ^a1^
	Day 3	5.51 ± 0.13 ^a1^	5.47 ± 0 ^a1^	5.47 ± 0.06 ^a1^	5.44 ± 0.04 ^a1^
	Day 4	5.61 ± 0.11 ^a1^	5.62 ± 0.16 ^a1^	5.6 ± 0.15 ^a1^	5.67 ± 0.23 ^a1^
	Day 5	5.75 ± 0.09 ^a1^	5.75 ± 0.2 ^a1^	5.73 ± 0.21 ^a1^	5.75 ± 0.21 ^a1^
Chicken	Day 1	4.95 ± 0.74 ^a1^	5.63 ± 0 ^a1^	5.44 ± 0.06 ^a1^	5.41 ± 0.02 ^a1^
	Day 2	5.55 ± 0.02 ^a,b1^	5.73 ± 0.04 ^b1^	5.5 ± 0.09 ^a,b1^	5.44 ± 0.06 ^a1^
	Day 3	5.7 ± 0.13 ^a1^	5.76 ± 0.01 ^a1^	5.64 ± 0.11 ^a1,2^	5.59 ± 0 ^a1,2^
	Day 4	5.78 ± 0.12 ^a1^	5.99 ± 0.26 ^a1^	5.77 ± 0.01 ^a2^	5.89 ± 0.14 ^a2^
	Day 5	5.86 ± 0.07 ^a1^	6.04 ± 0.25 ^a1^	5.83 ± 0.01 ^a2^	5.91 ± 0.11 ^a2^
Salmon	Day 1	6.11 ± 0.14 ^a1^	6.22 ± 0.05 ^a1^	6.2 ± 0.06 ^a1^	6.21 ± 0.01 ^a1^
	Day 2	6.21 ± 0.01 ^a1^	6.23 ± 0.05 ^a1^	6.22 ± 0.04 ^a1^	6.23 ± 0.03 ^a1^
	Day 3	6.28 ± 0.13 ^a1^	6.26 ± 0.05 ^a1,2^	6.24 ± 0.06 ^a1^	6.26 ± 0.01 ^a1^
	Day 4	6.34 ± 0.11 ^a1^	6.36 ± 0.01 ^a1,2^	6.27 ± 0.04 ^a1^	6.3 ± 0.03 ^a1,2^
	Day 5	6.4 ± 0.13 ^a1^	6.46 ± 0.09 ^a2^	6.31 ± 0.02 ^a1^	6.41 ± 0.05 ^a2^

Each value is the mean of two replicates per treatment day and type of treatment ± standard deviation (SD). For every type of microbe, different superscript letters in the same row indicate significant differences (*p* < 0.05) between storage conditions for the same storage day, and different superscript numbers in the same column indicate significant differences (*p* < 0.05) between storage day for the same storage condition.

## Data Availability

Data is contained within the article.
